# EMVC-2: an efficient single-nucleotide variant caller based on expectation maximization

**DOI:** 10.1093/bioinformatics/btad681

**Published:** 2023-11-14

**Authors:** Guillermo Dufort y Álvarez, Martí Xargay-Ferrer, Alba Pagès-Zamora, Idoia Ochoa

**Affiliations:** INCO, Facultad de Ingeniería, Universidad de la República, Montevideo 11300, Uruguay; SPCOM Group, Universitat Politècnica de Catalunya – BarcelonaTech (UPC), 08034 Barcelona, Spain; SPCOM Group, Universitat Politècnica de Catalunya – BarcelonaTech (UPC), 08034 Barcelona, Spain; Department of Electrical Engineering, Tecnun, University of Navarra, 20018 Donostia, Spain

## Abstract

**Motivation:**

Single-nucleotide variants (SNVs) are the most common type of genetic variation in the human genome. Accurate and efficient detection of SNVs from next-generation sequencing (NGS) data is essential for various applications in genomics and personalized medicine. However, SNV calling methods usually suffer from high computational complexity and limited accuracy. In this context, there is a need for new methods that overcome these limitations and provide fast reliable results.

**Results:**

We present EMVC-2, a novel method for SNV calling from NGS data. EMVC-2 uses a multi-class ensemble classification approach based on the expectation–maximization algorithm that infers at each locus the most likely genotype from multiple labels provided by different learners. The inferred variants are then validated by a decision tree that filters out unlikely ones. We evaluate EMVC-2 on several publicly available real human NGS data for which the set of SNVs is available, and demonstrate that it outperforms state-of-the-art variant callers in terms of accuracy and speed, on average.

**Availability and implementation:**

EMVC-2 is coded in C and Python, and is freely available for download at: https://github.com/guilledufort/EMVC-2. EMVC-2 is also available in Bioconda.

## 1 Introduction

Next-generation sequencing (NGS) has become indispensable in several fields, specially in personalized medicine. Analysis of NGS data normally starts with variant calling, which consists of identifying the differences or *variants* between the sequenced genome and a reference genome of the same species. Most common variants are in the form of single-nucleotide variants (SNVs) and short insertions or deletions (INDELs). SNVs and INDELs can have significant effects on individuals from a clinical setting perspective, e.g. higher risk of certain diseases, predisposition to certain types of cancer, or impact on drug metabolism and response. Thus, it is crucial to design efficient variant callers that accurately identify an individual’s variants.

Available variant callers are still not 100% accurate, and some suffer from high computational complexity. Here, we build upon the previously proposed SNV caller EMVC (Pagès-Zamora *et al.* 2022) to provide an efficient and accurate variant caller competitive with the state-of-the-art. The main improvements of EMVC-2 include the addition of a decision tree model to filter out incorrectly inferred variants, and a fast and efficient implementation of the algorithm that is publicly available and ready to use by the community.

## 2 Methods and experimental results

The EMVC-2 method presented here consists of two steps. Firstly, a set of SNV candidates is identified by the EMVC algorithm presented in our previous work (Pagès-Zamora *et al.* 2022), which solves the variant calling problem as an unsupervised multi-class ensemble classification task using the expectation–maximization (EM) based iterative approach (Dempster *et al.* 1997). Indeed, the EMVC algorithm is an ensemble classifier, meaning that it combines tags provided by numerous individual classifiers taking into account their reliability, which is inferred by the algorithm itself. Furthermore, EMVC is an unsupervised algorithm, as it operates without the need for labeled ground-truth data for training, and it is a multi-class classifier, as opposed to a binary one since it sorts the observed data into more than two classes. Briefly, EMVC proceeds as follows. For each locus or position in the genome, EMVC estimates the posterior probability of ten classes, each corresponding to one of the possible genotypes {AA,CC,GG,TT,AC,AG,AT,CG,CT,GT}. The class with the highest probability is selected. A position is marked as an SNV candidate if the decided genotype for that position differs from the reference in at least one nucleotide. Secondly, EMVC-2 uses a Decision Tree Classifier (DTC) (Pedregosa *et al.* 2011) to filter the untrue SNV candidates identified in the first step. A DTC is chosen as models based on DTs have been shown to discriminate well between true and false called variants in similar settings (Zhang and Ochoa 2020, Cooke *et al.* 2021). Moreover, we refrain from using more complex models such as neural networks due to overfitting concerns. The DTC takes a set of features for each SNV candidate as input and decides whether to keep the SNV or not. The selected features are the genotype; the depth, which is the number of reads that map to that position; the percentage of reads at that position with an alternate nucleotide, i.e. different from the reference; and the entropy of the class probability distribution estimated in the first step, where lower entropy means higher confidence in the selected class. The DTC is trained on a 15×-coverage pair-end Whole Genome Sequenced (WGS) dataset for which the set of true SNVs is known and assumed to be correct (see Supplementary Material). We use a DTC with a low tree depth of 5 to prevent overfitting and to capture general statistical patterns. The training results show that the class entropy and the percentage of alternate nucleotides at the position are the key features for filtering out untrue variants. See Supplementary Material for more details on the method.

The proposed method EMVC-2 is evaluated using a collection of public human NGS datasets with a coverage between 15× and 30× from different subjects for which a *ground truth* or set of true SNVs is available. Table 1 summarizes the characteristics of the datasets, such as coverage, subject ID, and size. Data availability information is detailed in the Supplementary Material.

**Table 1.** Recall, precision, and f1-score of SNV callers EMVC-2, GATK, Platypus, and Strelka2 on different human datasets.^a^

Dataset	Coverage	Subject ID	Size (GB)	EMVC-2			GATK			Platypus			Strelka2		
				Recall	Precision	f1-score	Recall	Precision	f1-score	Recall	Precision	f1-score	Recall	Precision	f1-score
*ERR262997*	30×	HG001	104	0.980	0.981	0.980	0.975	0.815	0.888	0.940	**0.993**	0.966	**0.985**	0.991	**0.988**
*NovaSeq*	25×	HG001	49	0.991	0.989	0.990	**0.994**	0.995	**0.995**	0.965	0.995	0.980	0.991	**0.999**	**0.995**
*Ashkenazim son*	25×	HG002	48	0.886	0.937	0.911	**0.913**	0.962	**0.937**	0.842	**0.983**	0.907	0.840	0.788	0.813
*pangenomics2*	30×	HG002	61	0.991	0.982	0.986	**0.993**	0.996	**0.994**	0.964	0.995	0.979	0.990	**0.999**	**0.994**
*pangenomics3*	30×	HG003	66	**0.991**	0.986	0.988	**0.992**	0.996	**0.994**	0.964	0.995	0.979	0.990	**0.999**	**0.994**
*pangenomics4*	30×	HG004	61	0.991	0.982	0.986	**0.993**	0.996	**0.994**	0.965	0.995	0.980	0.990	**0.999**	**0.994**
*Chinese Son*	15×	HG005	34	**0.971**	0.989	0.980	**0.971**	0.993	**0.982**	0.934	0.994	0.963	0.964	**0.993**	0.978
Average				0.971	0.978	**0.975**	**0.976**	0.965	0.969	0.939	**0.993**	0.965	0.964	0.967	0.965

a Best results are highlighted in bold.

EMVC-2 is compared with three state-of-the-art variant callers: GATK (McKenna *et al.* 2010), Platypus (Rimmer *et al.* 2014), and Strelka2 (Kim *et al.* 2018). Each tool is run with its default configuration. For GATK, its best practice pipeline is followed, which involves aligning the data to the corresponding reference using bwa (Li and Durbin 2009), marking duplicates and re-calibrating quality scores with Picard, and calling variants with the HaplotypeCaller in GVCF mode. Note that EMVC-2, Platypus, and Strelka2 only require the alignment as a preprocessing step, which is also done with bwa. See Supplementary Material for additional details and specific commands, and for a comparison to EMVC (Pagès-Zamora *et al.* 2022). As shown in Table 4 in the Supplementary Material, the proposed EMVC-2 method, which combines EMVC with a DTC, provides a higher f1-score than EMVC alone for all datasets considered.

The *hap.py* pipeline (Krusche *et al.* 2018) is used to compare the VCFs generated by each tool with the ground truth and to compute three measures of accuracy: precision, recall, and f1-score. Precision is the fraction of true variants among all the variants called by a tool. Recall is the fraction of true variants that are correctly called by a tool. F1-score is the harmonic mean of precision and recall, and it reflects the overall accuracy of a tool.

All experiments are conducted on a server with 80 64 bit x86 Intel Xeon CPUs, 503.5 GB of RAM memory, and CentOS Linux 7.7.1908; each tool is configured to run with the maximum number of threads.

The performance of each tool on the selected datasets is provided in Table 1. The results indicate that EMVC-2 is the most accurate tool on average across all the datasets, with an average f1-score of 0.975, followed by GATK with an average f1-score of 0.969. Platypus has the highest precision on average (0.993), but it is much less sensitive than the other tools. On another note, both GATK and Strelka2 achieve the highest number of individual best f1-score results across the datasets, but lack the consistency achieved by EMVC-2 to be the best on average.

**Figure 1.**
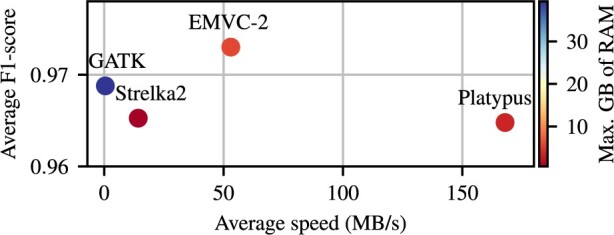
Average f1-score versus processing speed for EMVC-2, GATK, Strelka2, and Platypus.

The computational performance of the tools is assessed by measuring the processing speed, defined as the size of the dataset, in MB, divided by the time in seconds needed to obtain the variants after performing the alignment, and memory in GB required to process each dataset. In the experiments, Platypus was the fastest tool, with an average processing speed of 168 MB/s, followed by EMVC-2 with 53 MB/s, which was 3.7× times faster that Strelka2 and 106× times faster than GATK (see Fig. 1). Strelka2 was the most memory-efficient, using a maximum of 0.6 GB of RAM during the processing of the datasets, while EMVC-2, Platypus, and GATK, used a maximum of 6.0, 3.59, and 39.4 GB of RAM, respectively. A comparison with EMVC is omitted due to a lack of an efficient implementation.

In conclusion, EMVC-2 offers a great trade-off between SNV variant call accuracy and computational efficiency, thus adding to the available toolset of variant callers.

## Supplementary Material

btad681_Supplementary_Data

## References

[btad681-B1] Cooke DP , Wedge DC, Lunter G. A unified haplotype-based method for accurate and comprehensive variant calling. Nat Biotechnol 2021;39:885–92. 33782612 10.1038/s41587-021-00861-3 PMC7611855

[btad681-B2] Dempster AP , Laird NM, Rubin DB. Maximum likelihood from incomplete data via the EM algorithm. J R Stat Soc Ser B (Methodological) 1977;39:1–22.

[btad681-B3] Kim S , Scheffler K, Halpern A, et al Strelka2: fast and accurate calling of germline and somatic variants. Nat Methods 2018;15:591–4. 30013048 10.1038/s41592-018-0051-x

[btad681-B4] Krusche P , Trigg L, Boutros P et al; Global Alliance for Genomics and Health Benchmarking Team. Best practices for benchmarking germline small variant calls in human genomes. Nat Biotechnol 2018;37:555–60. 10.1038/s41587-019-0054-x PMC6699627 30858580

[btad681-B5] Li H , Durbin R. Fast and accurate short read alignment with Burrows–Wheeler transform. Bioinformatics 2009;25:1754–60. 19451168 10.1093/bioinformatics/btp324 PMC2705234

[btad681-B6] McKenna A , Hanna M, Banks E et al The genome analysis toolkit: a MapReduce framework for analyzing next-generation DNA sequencing data. Genome Res 2010;20:1297–303. 20644199 10.1101/gr.107524.110 PMC2928508

[btad681-B7] Pagès-Zamora A , Ochoa I, Cavero G et al Unsupervised ensemble learning for genome sequencing. Patt Recognit 2022;129:108721.

[btad681-B8] Pedregosa F , Varoquaux G, Gramfort A et al Scikit-learn: machine learning in Python. J Mach Learn Res 2011;12:2825–30.

[btad681-B9] Rimmer A , Phan H, Mathieson I et al; WGS500 Consortium. Integrating mapping-, assembly-and haplotype-based approaches for calling variants in clinical sequencing applications. Nat Genet 2014;46:912–8. 25017105 10.1038/ng.3036 PMC4753679

[btad681-B10] Zhang C , Ochoa I. VEF: a variant filtering tool based on ensemble methods. Bioinformatics 2020;36:2328–36. 31873730 10.1093/bioinformatics/btz952

