# Supplementary material for: EMVC-2: an efficient single-nucleotide variant caller based on expectation maximization
Source: Bioinformatics. 2023 Nov 14;40(3):btad681. doi: 10.1093/bioinformatics/btad681 (PMC10919945; doi:10.1093/bioinformatics/btad681)
Supplement: btad681_Supplementary_Data [file btad681_supplementary_data.pdf]

Supplementary data for manuscript "EMVC-2: An efficient single-nucleotide variant caller based on expectation maximization"

Guillermo Dufort y Álvarez[1], Martí Xargay-Ferrer[2], Idoia Ochoa*[3], and Alba Pagès-Zamora*[2]

[1]*INCO, Facultad de Ingeniería, Universidad de la República, Uruguay*
[2]*SPCOM Group, Universitat Politècnica de Catalunya - BarcelonaTech, Spain*
[3]*Department of Electrical Engineering, Tecnun, University of Navarra, Spain*

Contents

1 Method **2**

2 Implementation **5**

3 Data **5**
- 3.1 Ground truth VCF and bed files . . . 5
- 3.2 Datasets . . . 6
- 3.3 Human reference . . . 6

4 Tools and commands for experiments **7**
- 4.1 bwa and samtools . . . 7
- 4.2 EMVC-2 . . . 7
- 4.3 GATK . . . 7
- 4.4 Strelka2 . . . 9
- 4.5 Platypus . . . 9
- 4.6 Hap.py . . . 10

5 Extended results **10**
5.1 EMVC vs EMVC-2 10
5.2 Processing speed and memory consumption results 10
5.3 GATK results using Hard Filtering and Variant Quality Score Recalibration 11

1 Method

The developed EMVC-2 method consists of two steps. Firstly, a set of SNV candidates is identified by the algorithm EMVC presented in our previous work [5], which solves the variant calling problem as an unsupervised multi-class ensemble classification task using the expectation-maximization (EM) based iterative approach [2]. For each position or locus in the genome, this algorithm estimates the posterior probability of ten classes, each corresponding to one of the possible genotypes $\{AA, CC, GG, TT, AC, AG, AT, CG, CT, GT\}$. The class with the highest probability is selected. A position is marked as an SNV candidate if the decided genotype for that position differs from the reference in at least one nucleotide. Further details on this step can be found in [5].

The second step of the method uses a Decision Tree Classifier (DTC) [6] to filter the variant candidates identified by the EM-based algorithm of the first step. The goal is to filter out the untrue identified variants while retaining the true ones. The DTC uses four features to classify each variant: genotype (GT), depth (DP), alternative percentage (ALT_%), and class entropy (CLASS_Entropy). The definitions and calculations of these features are as follows:

- Genotype (GT): The genotype field of a variant in a VCF file shows the allele values separated by either "/" or "—". The allele values are 0 for the reference allele (REF) and 1 for the alternate allele (ALT). If more than one alternate allele if provided in ALT, 1 is used for the first allele, 2 for the second one, and so on. We generated an integer feature by concatenating the allele values, GT= X_1X_2, where X1/X2 is the VCF genotype.
- Depth (DP): The read depth at the considered position.
- Alternative percentage (ALT_%): The ratio of alternative alleles, given by $\frac{A}{DP}$, where A is the number of reads with ALT and DP is the total number of reads at that position.
- Class entropy (CLASS_Entropy): The entropy of the discrete probability distribution for the 10 possible classes estimated by the EM-based algorithm.

EMVC-2 uses the *DecisionTreeClassifier* from *sklearn.tree* with max_depth=5, min_samples_split=100, and min_samples_leaf=100. The DTC is trained on dataset ERR174324, from human HG001, for which the set of true SNVs is available, i.e., a ground truth exists. The dataset was aligned using bwa to reference GRCh37/b37 from the 1000 Genomes project. See below for download links

for the dataset, corresponding ground truth, and reference genome. After running the first step of EMVC-2, the considered features are computed for each identified variant, which are further labeled as true or false by comparing them with the ground truth. From the 330,241 identified variants, 316,948 were labeled as true and 13,293 as false.

The DTC hyperparameters were chosen via grid search as the ones that maximized the accuracy on the test set, with a 80/20 ratio for training and testing. Other features were also considered, such as chromosome number and position, probability of the selected genotype (computed by EMVC), probabilities of all genotypes, REF value, ALT value, strand bias, number of SNV candidates on a given window, among others, but no improvement on the results was observed. Once the hyperparameters were set, the DTC was trained with the entire data. The resulting tree, implemented in EMVC-2, is depicted in Figure 1. The DTC is applied to all SNV candidates identified in the first step of the algorithm, and only those classified as true are output in the VCF file.

The results of applying the DTC on the training data are shown in Table 1, and the feature importance in Table 2. From the 13,293 untrue variants, more than 10,000 variants are filtered out (77%), while only about 2% of true variants are lost. The overall accuracy increases from 95.97% to 97.15%. From the use features, the CLASS_Entropy is the most informative one, followed by ALT_%.

Table 1: Classification report of the considered DTC on the training data.

	Precision	Recall	F1-score	Support
FP	0.77	0.51	0.62	13293
TP	0.98	0.99	0.99	316948
Accuracy		0.97		330241
Macro avg	0.87	0.75	0.80	330241
Weighted avg	0.97	0.97	0.97	330241

Table 2: Feature importance.

Feature	Importance
CLASS_Entropy	0.576
ALT_%	0.221
DP	0.117
GT	0.086

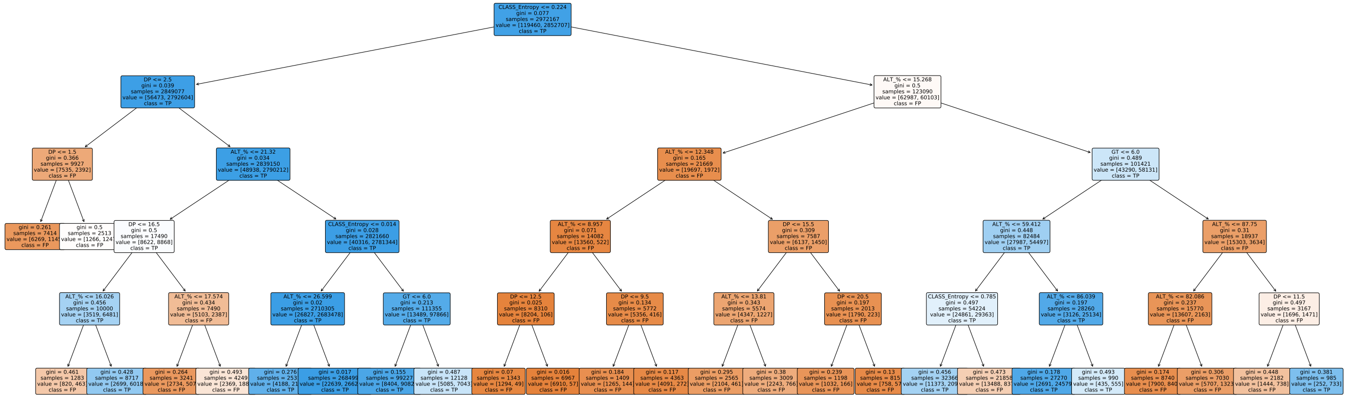

Figure 1: Decision Tree Classifier used by EMVC-2 to filter out untrue variants.

2 Implementation

The first step of EMVC-2 consists on the EMVC algorithm, introduced and fully described in [5]. However, the implementation was not made available, due to the lack of an efficient implementation ready to use by the community. In EMVC-2, the EMVC algorithm is implemented efficiently in C, and the DTC in Python. Moreover, the EMVC algorithm can be run in parallel, being able to analyze more than one chromosome simultaneously. EMVC-2 can be run with a single command.

The code is available at `https://github.com/guilledufort/EMVC`. We also provide all necessary scripts and instructions to run EMVC-2. Finally, the EMVC-2 algorithm has been deposited in the Bioconda channel and can be easily installed and used with Conda on Linux and MacOS.

3 Data

For the experiments, we used data from human individuals HG001, HG002, HG003, HG004 and HG005. These individuals have been fully characterized by the Genome in a Bottle Consortium (GIAB), a public-private-academic consortium hosted by NIST (`https://www.nist.gov/programs-projects/genome-bottle`), and a set of true SNVs, i.e., a ground truth, exists for each of them.

3.1 Ground truth VCF and bed files

The ground truth VCF files and corresponding confident regions bed files were downloaded for each human subject from these sources:

- **HG001:** `https://ftp-trace.ncbi.nlm.nih.gov/giab/ftp/release/NA12878_HG001/ChineseSonv4.2.1/GRCh37/`
- **HG002:** `https://ftp-trace.ncbi.nlm.nih.gov/giab/ftp/release/AshkenazimTrio/HG002_NA24385_son/ChineseSonv4.2.1/GRCh37/`
- **HG003:** `https://ftp-trace.ncbi.nlm.nih.gov/giab/ftp/release/AshkenazimTrio/HG003_NA24149_father/ChineseSonv4.2.1/GRCh37/`.
- **HG004:** `https://ftp-trace.ncbi.nlm.nih.gov/giab/ftp/release/AshkenazimTrio/HG004_NA24143_mother/ChineseSonv4.2.1/GRCh37/`
- **HG005:** `https://ftp-trace.ncbi.nlm.nih.gov/giab/ftp/release/ChineseTrio/HG005_NA24631_son/ChineseSonv4.2.1/GRCh37/`

The VCF and bed files refer to human reference GRCh37/b37.

3.2 Datasets

Next Generation Sequencing (NGS) data from the considered human subjects is used for our experiments. The datasets are summarized in Table 3.

Dataset	Reference	Size (GB)	Coverage	Sequencing Method
ERR262997	HG001	104	30	Illumina HiSeq 2000
NovaSeq	HG001	49	25	Illumina NovaSeq 6000
Ashkenazim son	HG002	48	25	Illumina HiSeq 2500
pangenomics2	HG002	61	30	Illumina HiSeq 2500
pangenomics3	HG003	66	30	Illumina HiSeq 2500
pangenomics4	HG004	61	30	Illumina HiSeq 2500
Chinese Son	HG005	34	15	Illumina HiSeq 2500

Table 3: Information of the different human datasets used for experimentation. * indicates that a public link is not available for download.

The NovaSeq dataset was generated with Illumina NovaSeq 6000. This dataset differentiates from the others by having an alphabet of only 4 different quality scores. The dataset was obtained from the MPEG-G standard (`https://mpeg-g.org/`) database which is not publicly available. The other datasets can be downloaded from the following links:

- **ERR262997:** `https://www.ebi.ac.uk/ena/browser/view/ERA207860?show=reads`
- **pangenomics2:** `https://s3-us-west-2.amazonaws.com/human-pangenomics/index.html?prefix=NHGRI_UCSC_panel/HG002/hpp_HG002_NA24385_son_v1/ILMN/downsampled/`
- **pangenomics3:** `https://s3-us-west-2.amazonaws.com/human-pangenomics/index.html?prefix=NHGRI_UCSC_panel/HG002/hpp_HG002_NA24385_son_v1/parents/ILMN/downsampled/HG003/`
- **pangenomics4:** `https://s3-us-west-2.amazonaws.com/human-pangenomics/index.html?prefix=NHGRI_UCSC_panel/HG002/hpp_HG002_NA24385_son_v1/parents/ILMN/downsampled/HG004/`
- **Chinese Son:** `https://ftp-trace.ncbi.nlm.nih.gov/giab/ftp/data/ChineseTrio/HG005_NA24631_son/NIST_Stanford_Illumina_6kb_matepair/fastqs/`

3.3 Human reference

All the datasets were aligned to human reference GRCh37/b37 from the 1000 Genomes Project, available at `https://ftp-trace.ncbi.nlm.nih.gov/giab/ftp/release/references/GRCh37/`.

4 Tools and commands for experiments

In this Section we detail all the tools and commands used for experimentation.

4.1 bwa and samtools

To align the datasets against the reference we use *bwa* version 0.7.17. The output file is sorted afterwards with *samtools* version 1.9, obtaining a BAM file. The following command is used:

```
bwa mem -t  [-@THREADS] [REF] [FASTQ_R1] [FASTQ_R2] \
| samtools sort [-@THREADS] -o [BAM_FILE]
```

4.2 EMVC-2

EMVC-2 receives two main parameters as input:

- **T**: The number of iterations of the EM algorithm.
- **M**: The number of learners used in the algorithm.

For our experiments we used $T = 5$ and $M = 7$, i.e, seven Q-score bins used by Illumina, which are the default values. We run EMVC-2 with the following command:

```
./emvc-2  \
-i [BAM_FILE] \
-r [REF] \
-p [@THREADS] \
-o [OUT_VCF]
```

4.3 GATK

For the Genome Analysis Toolkit (GATK) [4] we use version v4.3.0, with HTSJDK version 3.0.1 and Picard version 2.27.5, which can be downloaded from https://github.com/broadinstitute/gatk. To call the variants with GATK we followed the recommended best practices pipeline for Germline short variant discovery, available at https://gatk.broadinstitute.org/hc/en-us/articles/360035535932-Germline-short-variant-discovery-SNPs-Indels-.

To call variants for an input BAM_FILE, we run the following pipeline of commands:

```
[GATK_INSTALL_PATH]/gatk AddOrReplaceReadGroups \
        -I [BAM_FILE] \
        -O [RG_OUT] \
        -RGID 4 \
        -RGLB lib1 \
```

```
        -RGPL illumina \
        -RGPU unit1 \
        -RGSM 20

[GATK_INSTALL_PATH]/gatk MarkDuplicates \
        -I [RG_OUT] \
        -O [DEDUP_OUT] \
        -M [DEDUP_METRICS] \
        --REMOVE_DUPLICATES

[GATK_INSTALL_PATH]/gatk BaseRecalibrator \
-I [DEDUP_OUT] \
-R [REF] \
--known-sites [BED] \
-O [OUT_TABLE]

[GATK_INSTALL_PATH]/gatk ApplyBQSR \
-R [REF] \
-I [DEDUP_OUT] \
--bqsr-recal-file [OUT_TABLE] \
-O [OUT_BC]

[GATK_INSTALL_PATH]/gatk HaplotypeCaller \
--java-options \"-Xmx100G\" \
-R [REF] \
-I [OUT_BC] \
-O [OUT_VCF] \
--native-pair-hmm-threads [@THREADS]
```

For completion, GATK was run with two types of variant filtration methods: Hard Filtering (HF), and Variant Quality Score Re-calibration (VQSR). To perform the hard filtering we run the following command:

```
[GATK_INSTALL_PATH]/gatk VariantFiltration \
-R [REF] \
-V [IN_VCF] \
--filter-expression [FILTER_EXPRESSION] \
--filter-name "gatk_recommend_filter" \
-O [OUT_VCF]
```

where, FILTER_EXPRESSION was the default:

```
FILTER_EXP="QD < 2.0 || FS > 60.0 || MQ < 40.0 || MQRankSum < -12.5 || ReadPosRankSum < -8.0"
```

To perform VQSR we run the following commands with default configuration.

```
[GATK_INSTALL_PATH]/gatk VariantRecalibrator \
-R [REF] \
-V [IN_VCF] \
--resource:hapmap,known=false,training=true,truth=true,prior=15.0 vqsr/hapmap_3.3.b37.vcf \
--resource:omni,known=false,training=true,truth=true,prior=12.0 vqsr/1000G_omni2.5.b37.vcf \
--resource:1000G,known=false,training=true,truth=false,prior=10.0 vqsr\
/1000G_phase1.snps.high_confidence.b37.vcf \
--resource:dbsnp,known=true,training=false,truth=false,prior=2.0 vqsr/dbsnp_138.b37.vcf \
-an DP -an QD -an FS -an SOR -an MQ -an MQRankSum -an ReadPosRankSum \
--mode SNP -tranche 100.0 -tranche 99.9 -tranche 99.0 -tranche 90.0 \
-O [RECAL_FILE] \
--tranches-file [TRANCHES_FILE]

[GATK_INSTALL_PATH]/gatk ApplyVQSR \
-R [REF] \
-V [IN_VCF] \
--mode SNP \
--recal-file [RECAL_FILE] \
--tranches-file [TRANCHES_FILE] \
-ts-filter-level 99.5 \
-O [OUT_VCF]
```

4.4 Strelka2

We use the Strelka2 [3] variant caller version 2.9.10, which can be downloaded at `https://github.com/Illumina/strelka`. To run Strelka2, first we create a workflow script, "runWorkflow.py", for the input BAM_FILE:

```
[STRELKA_INSTALL_PATH]/bin/configureStrelkaGermlineWorkflow.py \
--bam [BAM_FILE] \
--referenceFasta [REF] \
--runDir [WORKFLOW_DIR]
```

Finally, we run the workflow script:

```
[WORKFLOW_DIR]/runWorkflow.py \
-m local \
-j [@THREADS]
```

4.5 Platypus

We use the Platypus [7] variant caller version 5.10.0, which can be downloaded from `https://github.com/andyrimmer/Platypus`. We run Platypus with the following command:

```
% Python 2
python [PLATYPUS_INSTALL_PATH]/bin/Platypus.py callVariants \
--bamFiles=[BAM_FILE] \
--refFile=[REF] \
--nCPU=[@THREADS] \
--genIndels=0 \
--output=[OUT_VCF]
```

4.6 Hap.py

In order to assess results of the different variant callers, we compared each tool VCF output against the ground truth of the dataset using the Hap.py pipeline [1] version 0.3.7, see `https://github.com/Illumina/hap.py`. We run the Hap.py pipeline with the following command:

```
[HAP.PY_INSTALL_PATH]/bin/hap.py \
[GROUND_TRUTH_VCF] \
[INPUT_VCF] \
-o [OUTPUT_NAME] \
-f [INPUT_BED_FILE] \
-r [REF] \
--engine vcfeval \
--preserve-info \
--pass-only \
--threads [@THREADS]
```

5 Extended results

5.1 EMVC vs EMVC-2

The original EMVC algorithm [5] and EMVC-2 are compared in Table 4. Note that the original EMVC algorithm was designed as a prototype in MATLAB and Python and was not intended to handle various scenarios. Therefore, we corrected minor bugs and added some functionalities to enable it to run on full BAM files with multiple chromosomes. Table 4 shows that EMVC-2 achieves higher f1-score than EMVC on all datasets. As expected, EMVC has higher recall, but much lower precision, which demonstrates the value of using the Decision Tree Classifier (DTC) to filter out the candidate variants.

5.2 Processing speed and memory consumption results

Table 5 shows the results of time and memory consumption of running each tool on each dataset. We omit results of the original EMVC due to the lack of an efficient implementation.

	Reference	Size (GB)	recall		precision		f1-score	
			EMVC	EMVC-2	EMVC	EMVC-2	EMVC	EMVC-2
ERR262997	HG001	104	**0.990**	0.980	0.958	**0.981**	0.974	**0.980**
NovaSeq	HG001	49	**0.994**	0.991	0.972	**0.989**	0.983	**0.990**
Ashkenazim son	HG002	48	**0.915**	0.886	0.844	**0.937**	0.878	**0.911**
pangenomics2	HG002	61	**0.994**	0.991	0.960	**0.982**	0.977	**0.986**
pangenomics3	HG003	66	**0.995**	0.991	0.967	**0.986**	0.980	**0.988**
pangenomics4	HG004	61	**0.995**	0.991	0.960	**0.982**	0.977	**0.986**
Chinese Son	HG005	34	**0.980**	0.971	0.973	**0.989**	0.976	**0.980**
		Average	**0.980**	0.971	0.948	**0.978**	0.964	**0.975**

Table 4: Performance of the original EMVC algorithm and the proposed EMVC-2 on the considered datasets. Best results are highlighted in bold.

	EMVC-2		GATK		Platypus		Strelka2	
	time (s)	mem (GB)	time (s)	mem (GB)	time (s)	mem (GB)	time (s)	mem (GB)
ERR262997	1226	4.1	224476	39.1	1011	2.8	6355	0.5
NovaSeq	2169	6.0	161931	38.4	309	3.6	5334	0.6
Ashkenazim son	801	4.4	137439	37.5	355	3.2	5154	0.4
pangenomics2	1715	4.4	117954	39.4	297	2.8	4418	0.6
pangenomics3	1995	5.9	112470	38.3	310	2.7	4463	0.6
pangenomics4	1454	4.2	113574	39.4	816	2.6	4673	0.5
Chinese Son	479	2.8	75445	37.8	157	2.3	1971	0.4

Table 5: Time (in seconds) and maximum memory consumption (in GB) of each tool in each dataset. GATK time includes preprocessing.

5.3 GATK results using Hard Filtering and Variant Quality Score Recalibration

Table 6 shows the results of running two types of variants filtering with GATK. The default configuration as specified in Section 4.3 was used for both filters.

	recall			precision			f1-score		
	None	HF	VQSR	None	HF	VQSR	None	HF	VQSR
ERR262997	**0.974**	0.956	0.948	0.831	**0.870**	0.835	0.897	**0.911**	0.888
NovaSeq	**0.994**	0.984	0.981	0.995	**0.998**	**0.998**	**0.994**	0.991	0.989
Ashkenazim son	**0.904**	0.885	0.873	0.964	0.967	**0.970**	**0.933**	0.924	0.919
pangenomics2	**0.991**	0.981	0.976	0.997	**0.998**	**0.998**	**0.994**	0.989	0.987
pangenomics3	**0.991**	0.980	0.976	0.996	**0.998**	0.997	**0.993**	0.989	0.986
pangenomics4	**0.992**	0.982	0.977	0.996	**0.998**	0.997	**0.994**	0.990	0.987
Chinese Son	**0.960**	0.947	0.937	0.992	**0.993**	**0.993**	**0.976**	0.969	0.964

Table 6: GATK results using no filter (none), hard filtering (HF), and Variant Quality Score Recalibration (VQSR).

Both HF and VQSR are shown to improve the precision results in each dataset consistently, at the cost of a lower recall, getting overall a considerably lower f1-score. In this sense, the best results in terms of f1-score are obtained by not applying any filter, and these results are reported in the main paper.

References

[1] James K Bonfield, John Marshall, Petr Danecek, Heng Li, Valeriu Ohan, Andrew Whitwham, Thomas Keane, and Robert M Davies. HTSlib: C library for reading/writing high-throughput sequencing data. *GigaScience*, 10(2), 02 2021. giab007.

[2] Rubin D.B. Dempster A.P., Laird N.M. Maximum Likelihood From Incomplete Data Via The EM Algorithm. *Journal Of The Royal Statistical Society: Series B (Methodological)*, 39(1):1–22, 1977.

[3] S. Kim, K. Scheffler, A. Halpern, M. Bekritsky, E. Noh, M. Källberg, X. Chen, Y. Kim, D. Beyter, P. Krusche, and Others. Strelka2: fast and accurate calling of germline and somatic variants. *Nature Methods*, 15:591–594, 2018.

[4] A. McKenna, M. Hanna, E. Banks, A. Sivachenko, K. Cibulskis, A. Kernytsky, K. Garimella, D. Altshuler, S. Gabriel, M. Daly, and Others. The genome analysis toolkit: a mapreduce framework for analyzing next-generation dna sequencing data. *Genome Research*, 20:1297–1303, 2010.

[5] A. Pagès-Zamora, I. Ochoa, G. Cavero, and P. Villalvilla-Ornat. Unsupervised ensemble learning for genome sequencing. *Pattern Recognition*, 129:108721, 2022.

[6] F. Pedregosa, G. Varoquaux, A. Gramfort, V. Michel, B. Thirion, O. Grisel, M. Blondel, P. Prettenhofer, R. Weiss, V. Dubourg, J. Vanderplas, A. Passos, D. Cournapeau, M. Brucher, M. Perrot, and E. Duchesnay. Scikit-learn: Machine learning in Python. *Journal of Machine Learning Research*, 12:2825–2830, 2011.

[7] A. Rimmer, H. Phan, I. Mathieson, Z. Iqbal, S. Twigg, W. Consortium, A. Wilkie, G. McVean, and G. Lunter. Integrating mapping-, assembly-and haplotype-based approaches for calling variants in clinical sequencing applications. *Nature Genetics*, 46:912–918, 2014.
